# Development, Urdu translation, and validation of a lifestyle modification manual for patients post-total knee replacement

**DOI:** 10.3389/fresc.2025.1565544

**Published:** 2025-04-29

**Authors:** Samreen Sadiq, Rabiya Noor, Hafiz Muhammad Asim

**Affiliations:** ^1^ Riphah College of Rehabilitation & Allied Health Sciences, Riphah International University, Lahore, Pakistan; ^2^ Department of Physical Therapy, Ghurki Trust Teaching Hospital, Lahore, Pakistan

**Keywords:** homecare, exercise, lifestyle modifications, total knee replacement, sensorimotor training

## Abstract

**Objective:**

Development, Urdu translation, and content validation of a manual on lifestyle modifications for individuals post-total knee replacement.

**Methods:**

A methodological study design was utilized in which a thorough literature review and a group interview with eight subjects were conducted, which led to the identification of domains and generation of items. The group consisted of 10 patients who had undergone total knee arthroplasty and five caregivers. A focal group discussion was conducted to evaluate the overlay and repetition among variable items. Two series of content validation were conducted by four content experts and four untrained individuals in English and Urdu. A revision was conducted for items with scores between 0.70 and 0.79 and retention was conducted for items that scored more than 0.79. Items with a content validity index score of less than 0.70 were omitted from the final set of items.

**Results:**

The initial review and focus group interview finalized 53 items in the exercise, education, and diet sections. After the discussion phase, the manual was tailored to 34 items. Content validation reported that the English version of the manual received validation scores of 0.864 and 0.858 for relevance and clarity, respectively. Likewise, the Urdu version of the manual attained Scale-Content Validity Index/Average scores of 0.864 and 0.900, respectively, for these metrics.

**Conclusion:**

A 34-item lifestyle modification manual was generated and its content was thoroughly validated to provide detailed guidance and instructions, enabling patients to continue their rehabilitation exercises and lifestyle modifications in the comfort of their own homes. The manual shows strong content validity, particularly for our target population in Pakistan.

## Introduction

Knee osteoarthritis (OA), along with low back pain, is one of the most frequent rheumatic disorders in Asian regions. A study conducted in Pakistan revealed that almost 28% of the urban and approximately 25% of the rural population are affected by knee OA (1). Total knee arthroplasty (TKA) is considered the gold standard management strategy for relieving symptoms due to degenerative arthritis of the knee. Rehabilitation after a knee replacement is an essential component among this population and plays a significant role in improving functional performance and quality of life (2). Rehabilitation after knee replacement focuses on the usual functional exercise training, ignoring a special type of therapeutic exercise, and considers not only isolated strengthening of a group of muscles but also improving central nervous system function to manage movement and appropriate muscular firing patterns to maintain joint stability, which is known as sensorimotor training (3).

Current novel trends post-knee replacement include strengthening training (4), a combination of neuromuscular electrical stimulation (NMES) with resistance training, the addition of sensorimotor components (5), and in-home telerehabilitation sessions post-total knee arthroplasty (6).

An important component in the treatment regime of patient with a knee replacement is the lifestyle modification program, which is a combination of education, exercise, and diet (7). Clinicians should update patients on the adverse features of unhealthy lifestyles and the advantages of engaging in healthy activities. Lifestyle modification programs endorse a low-calorie diet and exercise ranging from moderate to vigorous intensity for at least 60 min on most days (8). An initial assessment is needed to determine the current level of physical activity in individual patients. It is clinically useful to begin by asking patients how they judge their present level of physical activity and whether they believe that it is adequate to lose or maintain body weight. If, as usual, patients report being sedentary, the following step is asking why and whether there are physical or logistical barriers to exercise, e.g., arthritis or time constraints (9).

There is currently no standardized postoperative lifestyle modification manual (LMM) for total knee replacement (TKR) despite significant advancements in the field of total joint arthroplasty and lifestyle medicine. Despite the high prevalence of TKR in recent years, no home-based lifestyle change strategy is being properly implemented to address the muscular and functional deficits that result from surgery (10). Additionally, no studies have concentrated on evaluating the effects of these lifestyle modification protocols and these have not yet been examined in the Pakistani population. Therefore, an LMM was designed to address this need. This structured LMM focuses exclusively on modifiable health behaviors required to optimize the functional outcomes of patients at home following surgery. The LMM was created using the TKR rehabilitation concepts put forth in 2010 by Piva et al. (5). This study targets the development and formal content validation of an LMM for its applicability.

## Methodology

This study was conducted in the Orthopedic and Spine Center, Physiotherapy Department, Ghurki Trust Teaching Hospital, Lahore.

### Operational definitions

#### Lifestyle modification

Lifestyle modification is a significant step in rehabilitation among patients post-total knee replacement and requires promotion by healthcare specialists. It focuses on the combination of exercise, education, and diet (11).

#### Content experts

Experts involved in the pre-operative and operative procedures and rehabilitation of patients post-total knee replacement. Our study included an orthopedic surgeon, a physical therapist, a nutritionist, and a nurse.

#### Lay experts

Patients who have undergone primary total knee arthroplasty at Ghurki Trust Hospital.

### Demographics of the content experts and lay experts

The study involved both content experts and lay experts. The content experts included an orthopedic surgeon who was an expert in knee replacement surgeries, serving as a professor at the Orthopedic and Spine Center and actively performing knee surgeries. A physical therapist who specializes in musculoskeletal post-surgery procedures contributed, bringing over 27 years of experience in the field. The nutritionist held an M.Phil. degree and provided expertise in dietary management for post-surgical rehabilitation. The nurse, who was the head nurse in the orthopedic department, played a key role in patient care and recovery. The lay experts consisted of patients who had undergone primary total knee arthroplasty, offering firsthand insights into their rehabilitation journey. In addition, family members of patients participated in interviews to provide perspectives on caregiving and support during recovery.

### Development of the study manual

There were two stages of development of the LMM: (1) generation and development of the manual through a meticulous literature review and detailed interviews with the patients, preceded by a targeted group discussion; (2) the content validation and refinement of the manual by content experts and untrained individuals.

### Phase 1: designing the manual

A framework utilized in Iran was followed for the manual development and content validation that targets validation of tools developed with a patient centered approach (12). For content validation, in-depth interviews were conducted with 10 patients who underwent total knee arthroplasty along with five members of their families. Interview recording was conducted and the findings were transcribed. Afterward, the interviews were summarized by two team members. The family members’ inclusion was critical as arthroplasty impacts patients and family members play a significant role in their activities of daily living and care. This guaranteed a more complete understanding of the effects of TKA at the levels of patients and family caregivers. The group interview highlighted the apprehensions of patients after TKA and the rehabilitation aspects related to it. Three major sections were focused on as a result, including the type of exercises to be conducted at home, the diet plan to be followed, and an education section listing precautionary measures.

The manual items were formulated by combining results from the literature with findings from the group interview. Numerous databases and search engines were searched, including PubMed, Cochrane Library, and Google Scholar, using specific key terms such as “total knee arthroplasty”, “total knee replacement”, “total joint arthroplasty”, “life style modifications”, “behavior changes”, “exercise plan”, “home exercises”, “diet chart”, “education”, and “rehabilitation”. The literature showed different studies on home exercise plans, generic diet monitoring, and precautionary measures, whereas no studies were available on a structured home-based rehabilitation manual for patients after TKA. Consequently, various items were developed in each section by integrating the findings from the literature and the interviews with the patients and family members. On the basis of the insights gathered from the group interview and the existing evidence, a targeted group discussion was conducted to further explore the topic. The group consisted of two orthopedic surgeons, two research associates, a physical therapist specializing in musculoskeletal post-surgery procedures, a nutritionist, a nurse, and the patients and family members who had participated in the previous group interview.

Building upon the insights and responses collected from the targeted group discussion, a manual was developed. Special care was given to ensure the question items were phrased in a clear and straightforward manner, without any ambiguity. To cater to the local setting, the manual was also translated into Urdu by a professor from the Translation and Linguistic Department of the National University of Modern Languages (NUML), Islamabad, and a physical therapist. Furthermore, back-translating the Urdu version to English was conducted to verify the accuracy of the translation. The manual was first translated from English to Urdu by two independent bilingual experts. A back-translation was then performed by another set of bilingual professionals with expertise in medical and rehabilitation terminology. Any discrepancies were discussed and resolved through expert panel consensus to ensure conceptual equivalence.

### Phase 2: content validation of the manual

**Figure 1:**
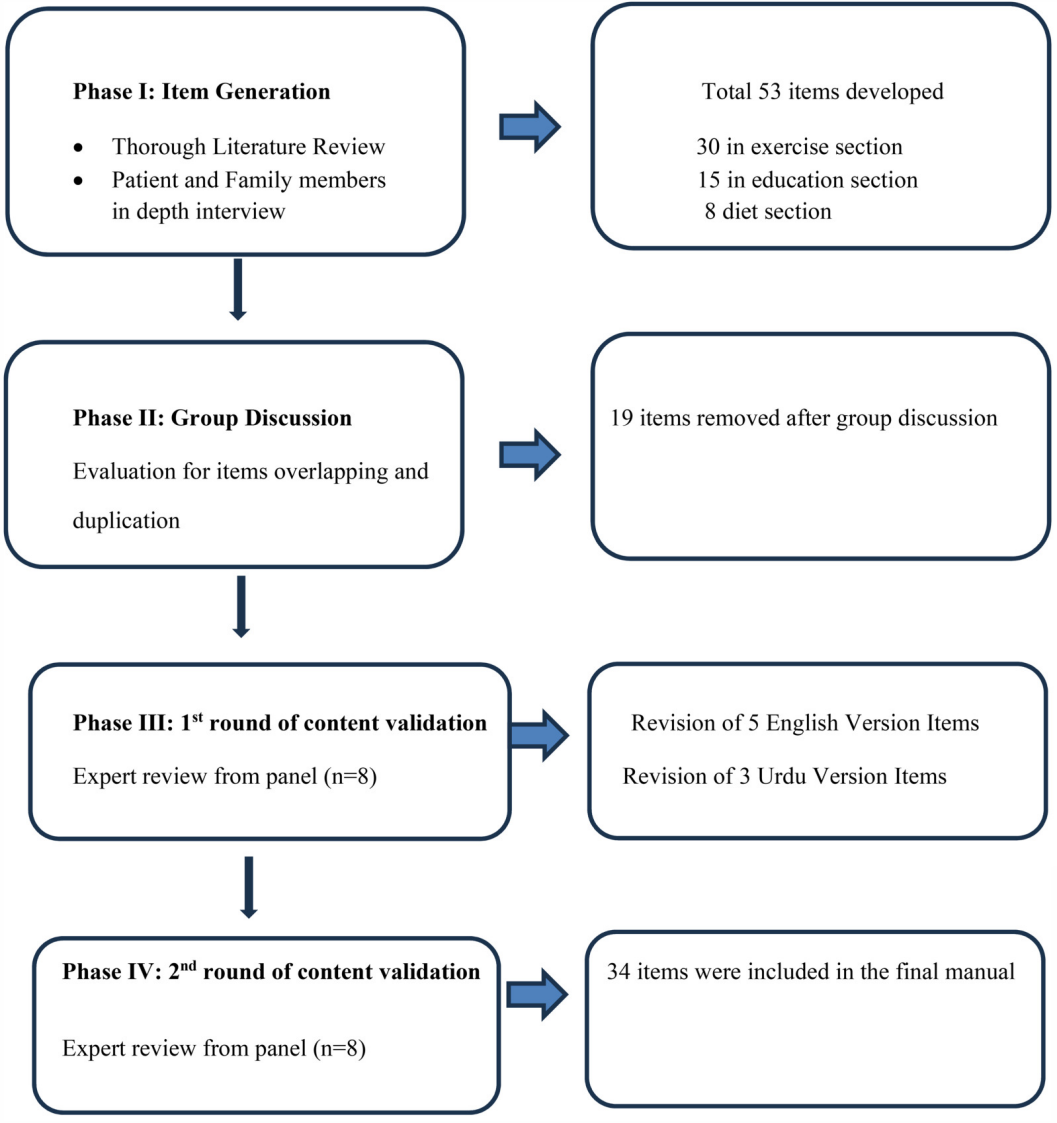
Flowchart depicting the process.

Content validation was performed to evaluate the clarity and relevance of the manual. The expert panel constituted four content and four lay experts. Both quantitative and qualitative judgments were given by the experts on manual items in English and Urdu. Special consideration was given to the selection of experts with bilingual proficiency and complete comprehension of both languages. Content experts were an orthopedic surgeon, physical therapist, nutritionist, and a nurse. The lay experts involved were four patients who had undergone TKA. The research team obtained written consent from the content and lay experts prior to the validation process. The panelists were asked to evaluate both aspects in terms of clarity and relevance of individual items in a questionnaire utilizing a 4-point scale. A score of 4 was considered as highly relevant/clear, 3 as quite relevant/clear but requires rephrasing, 2 as somewhat relevant/clear, and 1 as not relevant/clear, as shown in Table 1. A request was made by panelists to provide their valuable opinions and comments on manual items. The expert feedback was integrated into the manual items concerned and an item content validity index (I-CVI) score was calculated for each item. This score was determined by a number of specialists providing a rating of either “highly relevant/clear” or “quite relevant/clear but needs rephrasing,” divided by the total number of specialists. Item retention was ensured for items scoring greater than 0.79 and the items were reviewed, with items categorized as excellent with a score of more than 0.74, good with 0.60–0.74, and fair with a score between 0.40 and 0.59. Consequently, some questions were rephrased, leading to the formulation of the final version of the questionnaire. The calculation of the Scale-Content Validity Index/Average (S-CVI/Ave) for the entire tool was conducted by summing the I-CVI scores and dividing by the number of items. The development and content validation procedures are presented in Figure 1, which involved a detailed literature assessment and interviews with patients and caregivers. This initially generated a total of 30 exercise section items, 15 education items, and 8 diet domain items. Targeted discussions were carried out to scrutinize these items for overlapping and repetition, resulting in a final manual of 21 exercise section items, 7 education items, and 6 diet domain items.

**Table 1:** Rating system used by the experts to validate the LMM.

No.	Relevance	Clarity
1	Not relevant	Not clear
2	Needs some revision	Needs some revision
3	Relevant but needs some revision	Clear but needs minor revision
4	Very relevant	Very clear

### Sample selection criteria

The manual is applicable to both male and female patients who have undergone successful TKA, are aged between 45 and 75 years, have an active knee flexion of at least 80° and an extension lag of −10° upon discharge, and have Internet access in their residential area. These criteria ensured that the manual is relevant for patients undergoing structured rehabilitation.

### Patients and their family members' participation

The patients participated in the study as study subjects, while the family members helped in the formation and content validation of the manual. However, the procedure of content validation was the only one in which the content experts were involved. The final manual was created using input from lay experts and content experts. Every patient involved in the study, including the lay experts, had undergone TKA during the previous 6 months as a result of knee osteoarthritis.

Potential participants for the focus group interview were communicated with over the telephone, requested to contribute to the study, and provided an explanation of the goals of the investigation.

To identify the domains and generate items, five patients and three family caregivers consented to take part in the study. Following a prearranged group interview, the principal investigator received verbal consent from each participant before starting. Two team members conducted the transcription and summarized the findings after the interview sessions were recorded.

Patients were recruited to participate in the study once being informed of its goal. If they were in agreement, a clinic appointment was scheduled following their consultation. Before requesting that the patients complete the questionnaire, a written agreement was obtained from them. On a hard copy, patients were then asked to rate the items in the manual for relevance and clarity. To validate the content, the researchers reached out to content experts through telephone conversations. During these discussions, the specialists were given a summary of the study's aims and were asked to take part. Upon their acceptance, investigators forwarded a copy of the consent form and manual toward experts. Specialists were requested to evaluate the relevance and clarity of the materials. After finishing the evaluation, the experts communicated with researchers to submit a consent form and manual. The researchers replicated this procedure for the secondary phase of content validation, involving both levels, i.e., content experts and lay persons.

## Results

The CVI score results for both versions of the prefinal manual, based on relevance and clarity, are shown in Tables 2, 3. Our study's findings show that the majority of the items had good performance. Items that had a clarity and relevance CVI score greater than 0.78 were retained. Since every item received a score of 0.7 or better on both indices, none of the items were removed from the prefinal manual. For relevance and clarity, the prefinal English manual received S-CVI/Ave scores of 0.90 and 0.84, respectively. Similarly, the prefinal manual in Urdu had S-CVI/Ave scores of 0.91 and 0.94, respectively. Three items from the English version of the prefinal questionnaire and two items from the Urdu version of the prefinal questionnaire had scores between 0.70 and 0.79 for relevance or clarity. To make these items more relevant and clear, they were revised in accordance with the expert panel's recommendations. For instance, the item “Front lying knee bends with ankle weights?” was revised and a description was added to “Ankle weights between 2 and 5 lbs” in item 12 in the evening section of the exercise category. In a similar manner, item 3 was changed from one cup of mixed salad to the description of the salad (add cucumber, tomatoes, and cabbage, and then add some lemon juice and black pepper for taste) in the lunch section of the diet category. The second phase of validation was piloted on chosen items by the same group of specialists. Table 3 presents the results for these items. All of the revised items did well in both indices. To create the final version of the manual, the five revised items were combined with the remaining original manual items from the prefinal manual. For the final manual, a total score was determined based on relevance and clarity. For relevance and clarity, the final English manual yielded total S-CVI/Ave scores of 0.91 and 0.87, up from 0.90 and 0.85, respectively. Similarly, the S-CVI/Ave score for relevance for the Urdu questionnaire remained constant at 0.93 since no items were changed, while the score for clarity increased to 0.94 from 0.93, as shown in Table 4.

**Table 2:** CVI scores for the English version of the manual.

Sr. No.	Relevance (CVI)	Kappa (K)	Analysis	Clarity (CVI)	Kappa (K)	Analysis
Exercise items						
1	1.0	1.00	Element preserved	0.9	0.89	Element preserved
2	0.8	0.79	Element preserved	0.9	0.89	Element preserved
3	0.9	0.89	Element preserved	0.8	0.79	Element preserved
4	0.9	.89	Element preserved	0.8	0.79	Element preserved
5	0.8	.79	Element preserved	0.8	0.79	Element preserved
6	1.0	1.00	Element preserved	1.0	1.00	Element preserved
7	1.0	1.00	Element preserved	0.9	0.89	Element preserved
8	0.8	.79	Element preserved	0.9	0.89	Element preserved
9	0.8	.79	Element preserved	0.9	0.89	Element preserved
10	0.9	.89	Element preserved	0.9	0.89	Element preserved
11	0.8	.79	Element preserved	0.8	0.79	Element preserved
12	0.7	.66	Revision required*	0.8	0.79	Element preserved
13	0.9	.89	Element preserved	0.8	0.79	Element preserved
14	1.0	1.00	Element preserved	1.0	1.0	Element preserved
15	1.0	1.00	Element preserved	1.0	1.0	Element preserved
16	0.9	.89	Element preserved	0.9	0.89	Element preserved
17	0.8	.79	Element preserved	0.9	0.89	Element preserved
18	1.0	1.00	Element preserved	0.8	0.79	Element preserved
19	0.9	.89	Element preserved	0.8	0.79	Element preserved
20	0.8	.79	Element preserved	0.9	0.89	Element preserved
21	0.8	.79	Element preserved	0.9	0.89	Element preserved
Education items						
1	0.9	.89	Element preserved	0.8	0.79	Element preserved
2	1.0	1.00	Element preserved	0.8	0.79	Element preserved
3	0.8	.79	Element preserved	0.9	0.89	Element preserved
4	1.0	1.00	Element preserved	0.9	0.89	Element preserved
5	0.9	.89	Element preserved	0.8	0.79	Element preserved
6	0.9	.89	Element preserved	0.8	0.79	Element preserved
7	0.8	.79	Element preserved	0.7	0.66	Revision required*
Diet items						
1	1.0	1.00	Element preserved	0.9	0.89	Element preserved
2	1.0	1.00	Element preserved	1.0	1.00	Element preserved
3	0.8	.79	Element preserved	0.7	0.66	Revision required*
4	0.9	.89	Element preserved	1.0	1.00	Element preserved
5	0.9	.89	Element preserved	1.0	1.00	Element preserved
6	0.9	.89	Element preserved	0.9	0.89	Element preserved
S-CVI/Ave	0.864			0.858		

* Revision is required for that item.

**Table 3:** CVI scores for the Urdu version of the manual.

Sr. No.	Relevance (CVI)	Kappa (K)	Analysis	Clarity (CVI)	Kappa (K)	Analysis
Exercise items						
1	0.9	0.89	Element preserved	0.8	0.79	Element preserved
2	0.8	0.79	Element preserved	0.8	0.79	Element preserved
3	0.9	0.89	Element preserved	0.9	0.89	Element preserved
4	1.0	1.00	Element preserved	0.9	0.89	Element preserved
5	1.0	1.00	Element preserved	1.0	1.00	Element preserved
6	0.9	0.89	Element preserved	1.0	1.00	Element preserved
7	0.8	0.79	Element preserved	1.0	1.00	Element preserved
8	0.7	0.66	Revision required*	0.9	0.89	Element preserved
9	0.8	0.79	Element preserved	0.9	0.89	Element preserved
10	0.9	0.89	Element preserved	0.9	0.89	Element preserved
11	0.9	0.89	Element preserved	0.9	0.89	Element preserved
12	1.0	1.00	Element preserved	1.0	1.00	Element preserved
13	1.0	1.00	Element preserved	1.0	1.00	Element preserved
14	0.9	0.89	Element preserved	1.0	1.00	Element preserved
15	0.8	0.79	Element preserved	0.8	0.79	Element preserved
16	0.8	0.79	Element preserved	0.8	0.79	Element preserved
17	0.9	0.89	Element preserved	0.8	0.79	Element preserved
18	0.9	0.89	Element preserved	0.8	0.79	Element preserved
19	1.0	1.00	Element preserved	0.9	0.89	Element preserved
20	0.8	0.79	Element preserved	1.0	1.00	Element preserved
21	0.9	0.89	Element preserved	1.0	1.00	Element preserved
Education items						
1	0.8	0.79	Element preserved	0.8	0.79	Element preserved
2	0.9	0.89	Element preserved	0.9	0.89	Element preserved
3	0.8	0.79	Element preserved	0.9	0.89	Element preserved
4	0.8	0.79	Element preserved	1.0	1.00	Element preserved
5	0.9	0.89	Element preserved	0.8	0.79	Element preserved
6	1.0	1.00	Element preserved	1.0	1.00	Element preserved
7	1.0	1.00	Element preserved	0.9	0.89	Element preserved
Diet items						
1	0.8	0.79	Element preserved	0.9	0.89	Item retained
2	0.9	0.89	Element preserved	0.9	0.89	Item retained
3	0.9	0.89	Element preserved	0.8	0.79	Item retained
4	1.0	1.00	Element preserved	1.0	1.00	Item retained
5	0.8	0.79	Element preserved	0.7	0.69	Revision required*
6	0.9	0.89	Element preserved	1.0	1.00	Item retained
S-CVI/Ave	0.864			0.900		

* Revision is required for that item.

**Table 4:** CVI scores for the modified items in terms of relevance and clarity.

Sr. No.	Relevance (CVI)	Kappa (K)	Analysis	Clarity (CVI)	Kappa (K)	Analysis
Exercise items (English)						
12	1.0	1.00	Element preserved	1.0	1.00	Element preserved
Education items (English)						
7	1.0	1.00	Element preserved	1.0	1.00	Element preserved
Diet items (English)						
3	1.0	1.00	Element preserved	1.0	1.00	Element preserved
Exercise items (Urdu)						
8	1.0	1.00	Element preserved	1.0	1.00	Element preserved
Diet items (Urdu)						
5	1.0	1.00	Element preserved	1.0	1.00	Element preserved

Table 2 presents the CVI and Kappa (K) statistics for each item in the exercise, education, and diet sections of the LMM. The table assesses the relevance and clarity of each item based on expert evaluations.

• **Relevance (CVI):** Indicates the proportion of experts who rated the item as highly relevant or quite relevant. A score of ≥0.79 suggests the item is preserved, while a lower score may require revision.
• **Kappa (K):** Measures the level of agreement among experts beyond chance. A *K*-value close to 1.00 indicates strong agreement.
• **Clarity (CVI):** Reflects how clear and understandable each item was to the experts. Like relevance, a score of ≥0.79 suggests clarity is acceptable, whereas lower values may need modifications.
• **Analysis column:** Specifies whether an item was preserved (retained as is) or required revision based on expert feedback. Items marked as Revision Required* had lower clarity or relevance scores, indicating the need for improvement.
• **S-CVI/Ave (Scale-Content Validity Index/Average):** Represents the overall validity of the manual, calculated by averaging the CVI scores of all items. A value above 0.80 indicates strong content validity across the tool.

Overall, the results demonstrate that most items in the manual were preserved, with only a few requiring minor revisions to improve clarity or relevance.

Table 3 presents the CVI and K scores for the Urdu version of the LMM, evaluating the relevance and clarity of each item in the exercise, education, and diet sections.

• **Relevance (CVI):** Represents the proportion of experts who rated the item as highly relevant or quite relevant. Items with a score ≥0.79 were preserved, while lower scores indicated the need for revision.
• **Kappa (K):** Measures the level of agreement among the experts beyond chance. A *K*-value close to 1.00 indicates high agreement among experts.
• **Clarity (CVI):** Assesses how clearly each item was presented in the Urdu version. Items with scores ≥0.79 were considered clear, while lower scores required revision.
• **Analysis column:** Indicates whether an item was preserved (retained as is) or required revision based on expert feedback. Items marked as Revision Required* had lower clarity or relevance scores and needed modifications.
• **S-CVI/Ave (Scale-Content Validity Index/Average):** Represents the overall content validity of the Urdu version of the manual, with an average CVI score of 0.864 for relevance and 0.900 for clarity, indicating strong content validity.

Overall, the Urdu version of the manual demonstrated high validity, with most items being preserved and only a few requiring revisions for improved clarity.

## Discussion

A critical analysis was conducted to evaluate the relevance and clarity of a manual that provides guidance on lifestyle modifications specifically for the population undergoing knee replacement surgery in Pakistan. The primary objective of this study was to thoroughly assess the manual's content and its potential effectiveness.

We followed a rigorous methodology in developing and validating the manual, based on the approach outlined by Piva et al. (5). The initial items were created via comprehensive literature searching and interviews with patients and their family members. To prevent over-estimation of content validity, an expert committee was formulated, comprising eight participants, including four content experts and four lay experts. The inclusion of the lay experts ensured that the perspectives of the target population were adequately represented (13).

The revised questions underwent an additional round of content validation by the same panel of experts to ensure that all items were appropriately phrased and accurately represented the intended constructs they were designed to measure. This methodology of questionnaire development and validation is widely adopted and reported in various studies (14–17). While other studies have explored different strategies for questionnaire content validation (18, 19), we found the approach used in this study to be more rigorous and suitable for our study population.

The LMM was developed using a thorough approach with the aim of aligning its content to the specific needs and cultural background of Pakistani patients undergoing knee replacements. The content was developed to cover important lifestyle themes relevant to the target demographic, and the validation process involved incorporating valuable insights from healthcare experts. Some exercises were not taken into consideration by the group of experts who evaluated the face validity of the protocol prior to the validation process. This includes the strengthening of the knee rotators and encouraging knee extension with prone leg hang and NMES for the quadriceps. According to a Cochrane study from 2010, there is insufficient evidence to support the use of NMES in the advanced phase of rehabilitation after TKR (20).

Experts believed that the muscle activation program had a high agreement. Muscle activation programs will take care of those needs because muscle impairments are the most important parameter to be addressed after TKR (10). The manual goes into more detail about the benefits of resistance training and how to put it into practice. This stage also had a high validity score, as studies have shown that resistance training will produce superior results if muscular imbalances are corrected with muscle activation exercises (21).

The items in the education section also showed excellent agreement. The significance of education instructions in the form of videos, pamphlets, or apps has been indicated in the literature (22). The diet section was missing in the literature and not commonly provided to total knee arthroplasty patients. This study highlighted the importance of this component and added it to the manual.

A previous systematic review demonstrated that the addition of a structured lifestyle modification program to a traditional exercise regimen yields greater benefits for individuals with knee OA. A meta-analysis of randomized controlled trials showed that incorporating lifestyle modifications significantly reduces pain intensity, improves joint stiffness, and enhances physical function over 6 months. While individual studies have suggested improvements in quality of life, the meta-analysis did not confirm this effect (23).

Our study extends these findings by validating an LMM specifically tailored for patients who have undergone TKA. Unlike previous research focusing on OA management, our manual is structured to address post-surgical rehabilitation, integrating exercise, diet, and education to optimize recovery. The validation process confirmed the clarity and relevance of the manual items, emphasizing the need for a comprehensive rehabilitation approach beyond conventional exercise therapy.

## Strengths and limitations

The study has several notable strengths. This is the first of its kind within our population to create a manual that is specifically relevant and applicable to the unique circumstances of patients undergoing TKA. The manual aimed to comprehensively evaluate the lifestyle modifications associated with TKA. We followed an established framework of manual development and content validation proposed by Piva et al. (5). In addition, we ensured the involvement of all pertinent stakeholders, including subject matter experts and lay experts, providing us with a comprehensive and diverse perspective for manual development and validation.

The selected panel of experts possessed bilingual proficiency, allowing us to conduct content validation for both the English and local language versions of the manual using the same panel. This ensures the manual is accessible and applicable to our diverse population. In the future, our aspiration is to implement this manual on a broad scale, encompassing both private and public healthcare sectors, to gauge the prevalence of these challenges and offer guidance to clinicians on how to address them effectively. This will serve as the foundation for improving the long-term outcomes and quality of life for patients undergoing TKA.

Our study also has certain limitations. The results of the study are particular to Pakistani patients undergoing knee replacements. It is important to use caution when extrapolating the findings to different cultural or geographic contexts because healthcare practices and lifestyle preferences may differ. The results’ generalizability could be affected by the characteristics of the expert panel that participated in the content validation procedure. A more diversified panel might offer a more comprehensive viewpoint on the content validity of the manual. The choice of content experts for the validation procedure may have some intrinsic bias as they were chosen based on their experience in the field. The extent of the content validation may be limited if the experts have comparable viewpoints or experiences.

## Conclusion

A 34-item multifaceted manual was generated and rigorously validated to support both patients and clinicians in understanding how to use exercises, instructions, and diet to optimize home-based specific protocols for rehabilitation after knee arthroplasty. It can facilitate the implementation of evidence-based lifestyle modification approaches for this population by providing clinicians with a tool to better understand holistic strategies and to self-assess their clinical practice. The manual employed in this research has demonstrated strong content validity, particularly for the population under investigation. Future research studies could utilize this manual to carry out a pilot study.

## Data Availability

The original contributions presented in the study are included in the article/Supplementary Material, further inquiries can be directed to the corresponding author.
