# Supplementary material for: Development, Urdu translation, and validation of a lifestyle modification manual for patients post-total knee replacement
Source: Front Rehabil Sci. 2025 Apr 29;6:1565544. doi: 10.3389/fresc.2025.1565544 (PMC12069396; doi:10.3389/fresc.2025.1565544)
Supplement: Supplementary file 1 [file Datasheet1.pdf]

Supplementary File 1

Home Based Life style modification manual for Patients undergoing Total Knee Arthroplasty

Exercise Section		
**Morning Exercises**		
Exercise	Description	SET/Repetition
Hamstring stretch	wall	1 min
Calf Stretch	wall	1 min.
Quads stretch	Towel, door frame	2 min

**Afternoon exercises**		
Exercise	Description	SET/Rep.
Step up and forward 6 inches	2 large books 6 inches high or large cardboard 6 inches high	2/10
Calf raises single leg on a step with support	Stair or Step	1/10
Side step up 2 inches	1-2 books 2 inches	2/10
Lunges alternating legs	None	1/10
Wall sit	Wall	1 min.
Standing hamstring curls	Cable machine or resistance band	1/10

Kick backs	Cable machine or resistance band	1/10
Sit to stand single leg	Chair	1/10
Straight leg raises to 16 inches with ankle weights	None	1/10
**Evening exercises**		
Front lying knee bends with ankle weights	Ankle weight: 2-5 lbs	1/10
Squat single leg half with support	Chair, table	1/10
Single leg bridges	Equipment required: none	2/10
Balance cross over walking 10 feet	2 objects	5 mins
Balance Shuttle Walking	3 objects	5 min
Balance changing directions	Another person required	5 min
Hamstring stretch	wall	1 min
Calf stretch	wall	1 min.
Quads stretch	Towel, door frame	2 min

**Education Section**
Avoid Pain
Avoid swelling
Balance instructions
Stretching information
Walk fast
Preserve Knee mobility

Alternate walk fast with stationary bike			
Stationary bike	Stationary bike with seat low and moderate resistance making full revolutions 3 times / week	Stationary bike	30 min

**Diet Section**	
**BREAKFAST**	Drink water or lemonade (little warm water with lemon) before breakfast<br><br>Then<br><br>1 egg (most preferred boiled) with bran bread<br><br>With<br><br>Tea/Milk (low fat milk)<br><br>OR<br><br>Oatmeal with milk
**MID MORNING**	1 fruit of your choice OR<br><br>Fresh juice (not preserved) OR<br><br>½ cup fresh berries OR<br><br>Fruit smoothie (most preferable for weight loss) (use low-fat yogurt in it)
**Lunch**	1 cup mix salad (add cucumber, tomatoes, cabbage then add some lemon juice and black pepper for taste)<br><br>+<br><br>3oz grilled desi chicken fish/ meat<br><br>OR<br><br>½ cup vegetable curry/ legumes with 1 roti. (spinach is helpful in weight loss) with bean salad<br><br>Also add low fat yogurt in lunch (if u have taken fruit smoothie before then it is not necessary in lunch)

	if you want to have rice you can have ½ cup of boiled rice with gravy
**Evening**	Tea if preferred<br><br>With 1 or 2 cookies<br><br>Or)<br><br>Green tea (add cinnamon and lemon and instead of sugar use honey)
**Dinner**	Bean salad (kidney beans, chicky peas,peas)<br><br>½ vegetable curry or any legume with small size roti
**Bed time**	1 glass milk (only low fat)<br><br>Or<br><br>Almonds

**FOODS ALLOWED**	**FOODS TO AVOID**
Fresh vegetables	Alcohol
Oatmeals	High sugar foods
Low-fat dairy products	Fried foods
Anti-oxidants	Processed foods
Lemonade	High fat
Nuts mostly almonds	Broiler chicken
Fresh fruits	High-fat food/dairy products
